# Optimizing oxytocin LC-MS/MS sensitivity by choosing the right column

**DOI:** 10.1016/j.plabm.2021.e00254

**Published:** 2021-09-01

**Authors:** Mariëtte T. Ackermans

**Affiliations:** Endocrine Laboratory, Department of Clinical Chemistry, Amsterdam UMC, University of Amsterdam, the Netherlands

**Keywords:** Oxytocin, Liquid chromatography, Reversed phase, HILIC

## Abstract

**Objective:**

Sensitivity is often an issue in bioanalytical LC-MS/MS applications. Commonly investigated parameters to improve it include additives to mobile phase, derivatization and sample-preparation. The nature of the column, however, is not frequently evaluated.

**Design and Methods:**

The sensitivity is compared for 18 different reversed phase and 2 different HILIC columns using 2 different mobile phase compositions. Sensitivity was evaluated in terms of S/N for 1,5 pg oxytocin on column, using a scouting gradient.

**Results:**

The measured signal to noise ranged from 55 to 1473, indicating a substantial difference in sensitivity. The most sensitive columns were the Synergi Hydro RP (reversed phase) and the Atlantis HILIC (HILIC).

**Conclusions:**

This study shows that choosing the right column contributes to the sensitivity of the method.

## 1 Introduction

Today, liquid chromatography-mass spectrometry (LC-MS/MS) is widely used in clinical and pharmacological laboratories for bioanalytical applications, even as the gold standard method. Although it has lots of advantages, one of the main challenges for bioanalytical applications is sensitivity as in these applications, the concentration of the analyte is often low (pico-molar range), and/or only a small volume of sample is available.

In our lab, we are developing an LC-MS/MS method for the determination of oxytocin, a hormone and a neurotransmitter. Oxytocin is a promising neurobiological target protein for psychiatric research because it is involved in social behavior as well as in the regulation of stress and emotional responses. Its determination in biological samples is, however, very challenging [1]. A number of different immunoassays are commercially available, but despite the many advantages, they have some important limitations. For example, Szeto et al. [2] show that cross-reactivity with various immuno-reactive substances present in biological samples plays an important role in immunoassays for the determination of oxytocin. Liquid chromatography is an alternative method [[3], [4], [5]]. Although these methods appear promising and solve several limitations of the immunoassay, their sensitivity is barely sufficient.

There are several options to enhance the sensitivity of LC-MS/MS in bioanalytical applications. Besides the improvement of the mass spectrometer in itself, common strategies include sensitivity enhancement through additives to the mobile phase or derivatization [[6], [7], [8], [9], [10]], by manipulating injection or focusing [11,12], or by optimizing sample pre-treatment [[13], [14], [15]].

One of the aspects affecting the sensitivity that has not so much been investigated, however, is the nature of the LC column. The choice of the LC column is usually accounted for based on characteristics such as the separation power needed, the desired run-time, compatibility with the sample pre-treatment, and perhaps even on the columns that are available in the lab. Less attention is paid to how the type of reversed phase or HILIC column material adds to sensitivity. The aim of this study was to investigate this influence on the sensitivity of the determination of the model compound oxytocin in LC-MS/MS.

## 2 Materials and methods

### 2.1 Chemicals

Oxytocin acetate was obtained from Bachem (art.nr 4016373, BACHEM, Bubendorf, Switzerland). ULC/MS –CC/SFC grade acetonitrile (art. nr. 0001204101), ULC/MS –CC/SFC methanol (art. nr. 0013684102) and Formic acid 99% (art. nr. 0006914143) were purchased at Biosolve (Valkenswaard, The Netherlands). Ammonium formate (art. nr. 156264) was purchased at Sigma-Aldrich (Zwijndrecht, The Netherlands). UPLC grade water was delivered by a Pure Flex system from Elga (Veolia Water Solutions & Technologies, Ede, The Netherlands).

### 2.2 LC columns

18 Reversed phase (RP) columns and 2 HILIC columns were tested. Table 1 shows their brand, type and dimensions.

**Table 1:** Tested LC columns.

Reversed Phase columns			
Nr.	Brand	Type	Dimensions
1	Waters	HSS PFP	2.1 × 50 mm, 1.8 μm
2	Waters	BEH C18	2.1 × 50 mm, 1.7 μm
3	Waters	CSH C18	2.1 × 50 mm, 1.7 μm
4	Waters	Cortecs C18+	2.1 × 50 mm, 1.7 μm
5	Waters	HSS T3	2.1 × 50 mm, 1.8 μm
6	Waters	BEH C4	2.1 × 50 mm, 1.7 μm
7	Waters	BEH Phenyl	2.1 × 50 mm, 1.7 μm
8	Phenomenex	Synergi Fusion-RP	2.0 × 50 mm 4 μm
9	Waters	CSH Fluoro-Phenyl	2.1 × 50 mm, 1.7 μm
10	Waters	Xbridge C8	2.1 × 50 mm, 2.5 μm
11	Phenomenex	Kinetex Biphenyl	2.1 × 50 mm, 1.7 μm
12	Phenomenex	Kinetex F5	2.1 × 100 mm 2.6 μm
13	Phenomenex	Kinetex C18	2.1 × 75 mm 2.6 μm
14	Agilent	Zorbax SB8	2.1 × 100 mm 1.8 μm
15	ThermoFischer	Hypersil Gold	2.1 × 30 mm 1.9 μm
16	Phenomenex	Synergi Polar RP	2.0 × 75 mm 4 μm 80 Å
17	Phenomenex	Synergi Hydro RP	2.0 × 100 mm 4 μm 80 Å
18	Phenomenex	Luna Phenyl-hexyl	2.0 × 100 mm 3 μm

HILIC columns			
Nr.	Brand	Type	Dimensions
19	Waters	BEH Amide	2.1 × 100 mm 1.7 μm
20	Waters	Atlantis HILIC	2.1 × 100 mm 3 μm

### 2.3 Oxytocin standard solution

Oxytocin acetate was dissolved in H_2_O at a concentration of 0.5 mg/mL. This stock was aliquoted in low protein bind Eppendorf tubes and kept at -80 °C. For the experiment, a dilution was made of 500 pg/mL in 10% acetonitrile with 0.1% formic acid for reversed phase experiments and in acetonitrile with 0.1% formic acid for HILIC experiments.

### 2.4 Liquid chromatography- mass spectrometry

Liquid chromatography was carried out on an Exion LC system (Shimadzu Benelux, ‘s-Hertogenbosch, The Netherlands). Column temperature was set to 30 °C. Flow was 0.400 mL/min. Injection volume was 10 μL. Mobile phase composition and gradient are given in Table 2.

**Table 2:** Instrumental details for liquid chromatography.

	Reversed phase			HILIC		
Combination 1:						
Mobile phase A	0.1% formic acid in H_2_O			0.1% formic acid in H_2_O		
Mobile phase B	0.1% formic acid in acetonitrile			0.1% formic acid in acetonitrile		
		
**Combination 2:**						
Mobile phase A	10 mM ammoniumformate in H_2_O			100 mM ammonium formate in H_2_O		
Mobile phase B	0.1% formic acid in acetonitrile			5:95 (v/v) 100 mM ammonium formate in H_2_O: acetonitrile. pH 3		
		
**Gradient**	*Time (min)*	*%A*	*%B*	*Time (min)*	*%A*	*%B*
	Initial	95	5	initial	0	0
	1.00	95	5	1.00	0	0
	8.00	60	40	8.00	35	65
	8.10	95	5	8.10	0	100
	10.00	95	5	10.00	0	100

Mass spectrometry was carried out on a Sciex 7500 Triple quadrupole mass spectrometer (Nieuwerkerk aan den IJssel, The Netherlands) operated in de MRM ESI + mode, spray voltage 2000 V. Ion source gas 1 and 2: 40 psi; curtain gas 32 psi, CAD gas 12. Temperature 450 °C. Q1 and Q3 unit resolution, pause time 5 ms, high mass cooling time 600 ms. Dwell time 250 ms, Entrance potential 10.0 V, Collision cell exit potential 15.0 V. Oxytocin quantifier MRM: 1007.4 > 723.2 (collision energy 42.0 V).

The instrument was operated under Sciex OS software (version 2.1.0.55343).

### 2.5 Experimental setup

For each column 5 consecutive injections of 500 pg/mL oxytocin were performed with mobile phase combination 1 and mobile phase combination 2 respectively. For the comparison of the columns the average value (calculated in excel) of the last 3 injections was used. Values compared were peak area, signal to noise (S/N), plate number and retention time.

## 3 Results

**Fig. 1:**
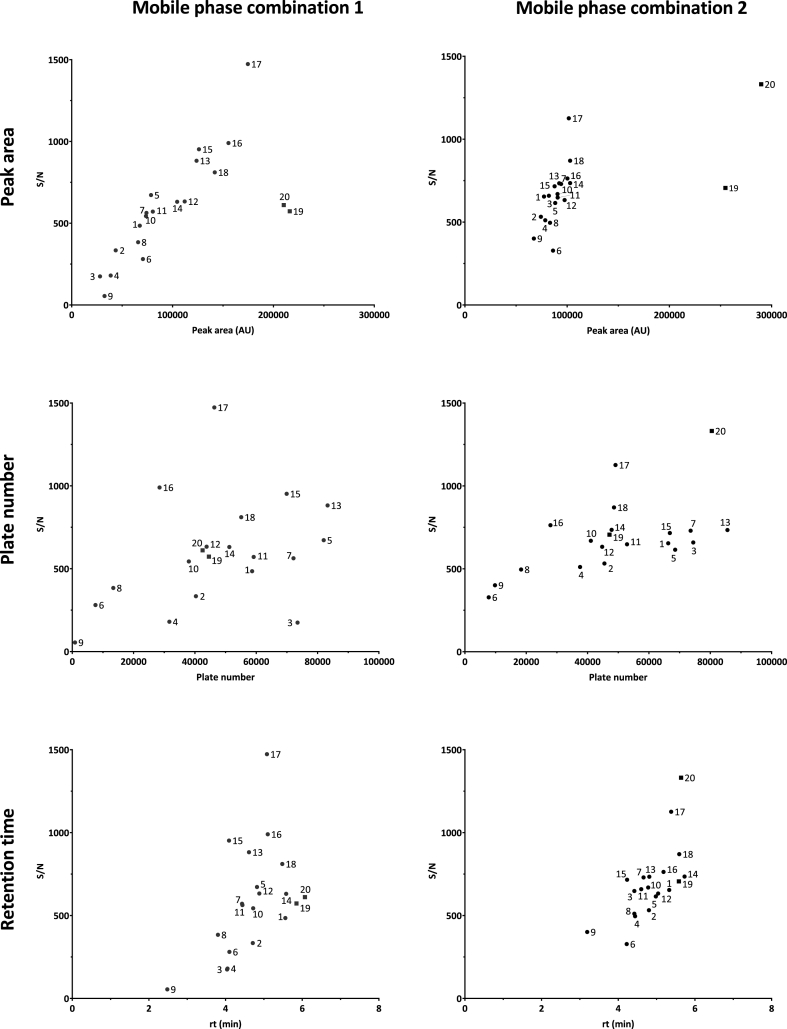
Signal to noise versus peak area, plate number and retention time for the different columns. (A) mobile phase combination 1; (B) mobile phase combination 2. Numbers refer to column numbers in Table 1.

Fig. 1 illustrates the S/N versus peak area, plate number, and retention time for the different columns and mobile phase conditions.

As expected, a positive correlation is seen between S/N and peak area, plate number and retention time. For RP columns, the difference between the columns is smaller using mobile phase condition 2 compared to mobile phase condition 1. As Fig. 1 illustrates, changing the “salt” composition of the mobile phase, results in a change in S/N. Compared to mobile phase combination 1, using mobile phase combination 2 some RP columns, for instance 3, 4, and 9, show higher peak area and S/N, while others such as 16, 17, and 18 show lower values. In some cases (e.g. column 9, mobile phase condition 1) a lower peak area is accompanied by a lower plate number, while in other cases (e.g. columns 5 and 7, mobile phase condition 1) a lower peak area is accompanied with a higher plate number. The effect on plate number between the two mobile phase conditions for most columns is less pronounced. Largest influence is found for the Altantis HILIC column (nr 20). The range in retention time is rather narrow. Columns where oxytocin has a similar retention time may show very different peak area (e.g. column 6 and 15, mobile phase condition 1) and consequently S/N. For RP separations column 17 with mobile phase condition 1 gives the best sensitivity. For HILIC separations this is the case for column 20 with mobile phase condition 2.

## 4 Discussion

The sensitivity of a bioanalytical method in LC-MS/MS depends on several parameters. On the basis of oxytocin as model compound, the study in this paper shows the chemistry of the LC column can play a role in achieving the desired sensitivity.

The sensitivity in LC-MS/MS, expressed as S/N, is mostly determined by ionization efficiency. In general, S/N increases with increasing peak area and/or plate number, which is illustrated in Fig. 1. Compared to the difference in peak area between the two mobile phase conditions, the difference in plate number is small. This finding suggests that the composition of the mobile phase in this case has more impact on ionization than on chromatography.

In addition, the percentage organic solvent in the mobile phase at the time of ionization has an influence on the efficiency of ionization and consequently sensitivity. In this study, retention times (except for column 9) are between 4 and 6 min and the difference in retention time for each column is limited in between the two mobile phase combinations. The fact that retention time is similar for each column between the two mobile phase compositions rules out the percentage of organic solvent as major cause for the better ionization and hence S/N. This is demonstrated by comparing for instance column 14 and 15 in mobile phase combination 1. Although the retention time for column 14 is higher, the peak area and the S/N are lower than for column 15.

A factor not so commonly evaluated with respect to sensitivity is the choice of the LC column. This choice is usually based on the nature of the analyte, the separation power needed, and the compatibility of the LC conditions with the sample pre-treatment. This study shows, however, that once the choice based on these parameters is made, it is worthwhile to test different columns with the same chemistry with respect to the sensitivity. For instance, comparing the columns with C18 chemistry (2, 3, 4, 5, 8, 13, 15, and 17) a huge difference in S/N is found, an effect that is even more pronounced using mobile phase condition 1 compared with 2. As a result, it is clear that using column 17 will result in a much more sensitive method compared to using, for instance, column 3. A possible explanation for this phenomenon is the bleeding of the column, which may on one the hand result in a higher noise level and in the other hand in ionization suppression of the compound of interest.

The columns tested in this study were the columns present in our lab. Not all of them were brand new, but they all showed a reasonably good peak shape. This suggests the column was still intact, however, it is possible that a new version of the column might give slightly different results.

## 5 Conclusion

In conclusion, this paper shows that columns might give substantially different sensitivities even if they are from the same chemistry. Choosing the right LC column and mobile phase conditions can increase sensitivity of the oxytocin LC-MS/MS method with several factors of magnitude. Although this study used oxytocin as model compound, the results most probably hold for others analytes, implying that when sensitivity of the method is an issue, choice of the column is important.
